# Large low field magnetocaloric effect in first-order phase transition compound TlFe_3_Te_3_ with low-level hysteresis

**DOI:** 10.1038/srep34235

**Published:** 2016-09-29

**Authors:** Qianhui Mao, Jinhu Yang, Hangdong Wang, Rajwali Khan, Jianhua Du, Yuxing Zhou, Binjie Xu, Qin Chen, Minghu Fang

**Affiliations:** 1 Department of Physics, Zhejiang University, Hangzhou 310027, China; 2 Department of Physics, Hangzhou Normal University, Hangzhou 310036, China; 3 Collaborative Innovation Center of Advanced Microstructures, Nanjing 210093, China

## Abstract

Magnetic refrigeration based on the magnetocaloric effect (MCE) is an environment-friendly, high-efficiency technology. It has been believed that a large MCE can be realized in the materials with a first-order magnetic transition (FOMT). Here, we found that TlFe_3_Te_3_ is a ferromagnetic metal with a first-order magnetic transition occurring at Curie temperature *T*_*C*_ = 220 K. The maximum values of magnetic entropy change (Δ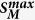) along the crystallographic *c*-axis, estimated from the magnetization data, reach to 5.9 J kg^−1^K^−1^ and 7.0 J kg^−1^ K^−1^ for the magnetic field changes, Δ*H* = 0–1 T and 0–2 T, respectively, which is significantly larger than that of MCE materials with a second-order magnetic transition (SOMT). Besides the large Δ*S*_*M*_, the low-level both thermal and field hysteresis make TlFe_3_Te_3_ compound an attractive candidate for magnetic refrigeration. Our findings should inspire the exploration of high performance new MCE materials.

Magnetic refrigeration based on MCE is an environment-friendly, high-efficiency technology compared to the traditional gas-cycle refrigeration^1^,^2^,^3^,^4^. After the discovery of the first magnetic refrigeration prototype near room temperature^5^ and the giant MCE in Gd_5_(Si_2_Ge_2_)^6^,^7^, a large MCE has been realized in a lot of materials in the past two decades, such as ReCo_2_ (Re = Er, Ho, and Dy) alloys^8^,^9^, manganite oxides (Re, M)MnO_3_ (Re = Lanthanide, M = Ca, Sr, and Ba)^10^,^11^, Ni-Mn-X (X = Ga, In, and Sn) based Heusler alloys^12^,^13^,^14^,^15^,^16^, MnAs based compounds^3^,^17^,^18^,^19^, La(Fe, Si)_13_ and related compounds^20^,^21^,^22^,^23^, as well as rare earth based intermetallic compounds^24^,^25^,^26^,^27^,^28^,^29^,^30^. Amongst the families of MCE materials, the compounds with first-order magnetic transition (FOMT) have been found promising due to their large and/or sharp changes in magnetization and the strong coupling between crystallographic structure and magnetism, such as Gd_5_Ge_4−*x*_Si_*x*_^7^, MnAs_1−*x*_Sb_*x*_^18^,^31^, MnFe(As, P, Si, Ge)^17^,^32^, LaFe_13−*x*_Si_*x*_(H_*δ*_)^14^,^21^,^23^ and Heusler-type magnetic shape-memory alloys^14^,^16^. However, in these materials, the magnetic transitions are frequently accompanied by significant thermal and/or magnetic hysteresis, which would limit the life span of refrigerants or even make the refrigeration cycle impossible^3^,^33^. In order to reduce or even eliminate the magnetic hysteresis losses, there have been two strategies. One is to treat the giant MCE materials by special methods, such as microstructure-tuning, as porosity^34^, fragmentation^35^,^36^,^37^, melt-spun^38^,^39^,^40^, or chemical tuning, as doping^41^. Another is to search for new high performance compounds with SOMT^42^. However, the performances in materials with SOMT are rather modest when compared with that with FOMT. It is therefore interesting to search for new FOMT materials with low-level hysteresis and without any additional treatments.

The crystal structure and physical properties of TlFe_3_Te_3_ were reported by two groups in 1984^43^,^44^. TlFe_3_Te_3_ crystalizes in a hexagonal structure with space group P6_3_/m, which consists of one-dimensional metallic cluster |Fe_3_Te_3_|_∞_ chains along the hexagonal *c*-axis, separated by the parallel chains of Tl atoms (see Fig. 1). The authors concluded that the compound undergoes a first-order transition from paramagnetic to ferromagnetic at 220 K based on their physical property measurements. However, neither of them observed discernible thermal and field hysteresis. Since the absence of hysteresis is appealing for magnetic refrigerant, in this report, we recheck the type of the magnetic transition and elucidate the MCE of TlFe_3_Te_3_ by performing resistivity and magnetization measurements. We found that this compound exhibits a large MCE with a small magnetic field change, Δ*H*, and with a low-level thermal and field hysteresis, thus identifying it to be another class of solids for the magnetic refrigerants.

## Results and Discussion

**Figure 1:**
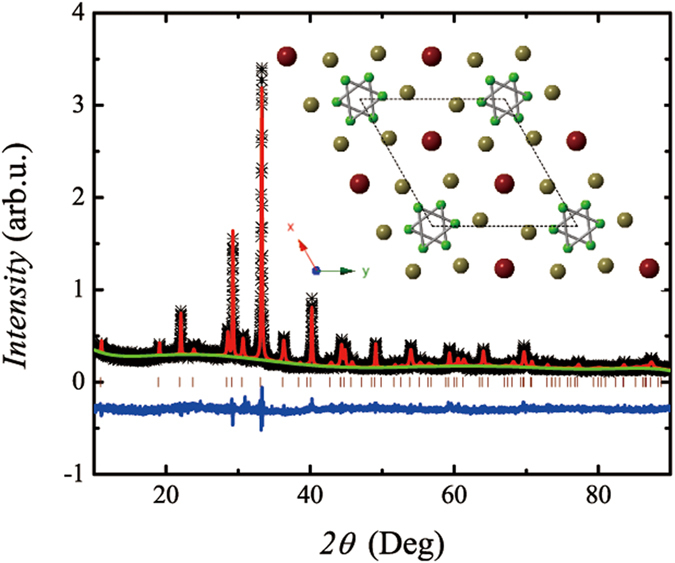
The powder X-ray diffraction (XRD) pattern (black star: observed data; red line: calculated curve; green line: background; blue line: difference; wine bar: Bragg positions) and the crystal structure of TlFe_3_Te_3_ viewed along *c*-axis (red ball: Tl; dark yellow ball: Te; green ball: Fe).

**Figure 2:**
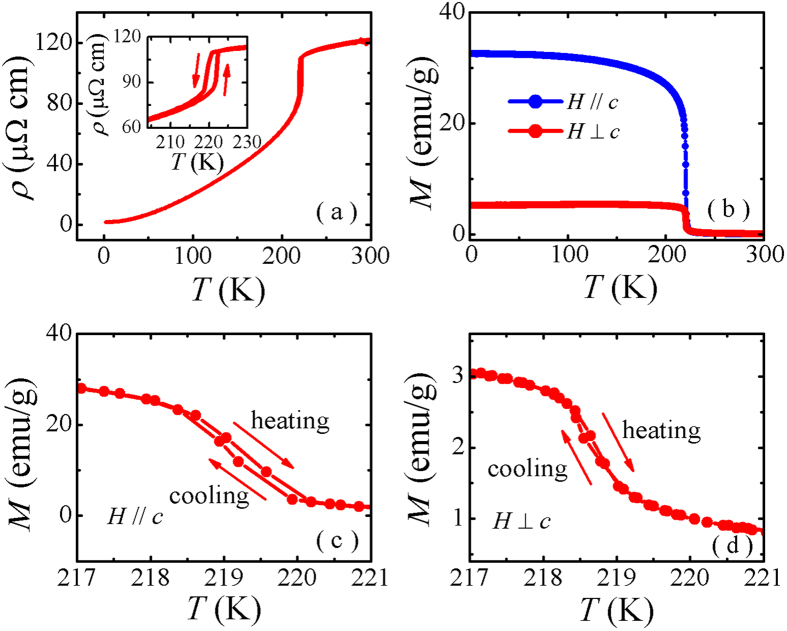
(**a**) The temperature dependence of resistivity with a current applied parallel to *c*-axis and the expansion near the transition temperature (inset). (**b**) The temperature dependence of magnetization, *M*(*T*), for both *H* || *c*-axis and *H* ⊥ *c*-axis. The *M*(*T*) near the transition temperature for (**c**) *H* || *c*-axis, (**d**) *H* ⊥ *c*-axis, the arrows show the cooling and heating process during measurements.

Figure 1 presents the powder x-ray diffraction (XRD) pattern of TlFe_3_Te_3_ and its Rietveld refinement. All the diffraction peaks could be indexed by a hexagonal structure with space group P6_3_/m. The lattice parameters *a* = 9.355(1) Å and *c* = 4.224(5) Å were obtained by the refinement, which are in good agreement with previous reports^44^. The electron probe micro-analyzer (EPMA) experiments performed on several single crystals verified that the sample composition (the average atomic ratio) is of Tl : Fe : Te = 0.99(1) : 2.95(2) : 3.00(1), which is in consistent with the nominal composition. The temperature dependence of electrical resistivity along *c*-axis, *ρ*(*T*), for a TlFe_3_Te_3_ crystal is shown in Fig. 2(a). In the whole measuring temperature range, the positive resistivity-temperature coefficient of *ρ*(*T*) indicates its metallic behavior. The resistivity has a very sharp drop at 220 K with detectable thermal hysteresis [see the inset of Fig. 2(a)], which is associated with the first-order ferromagnetic transition. The resistivity at 300 K and 1.8 K are of 120 *μ*Ω cm and 1.8 *μ*Ω cm, respectively. The small resistivity should be viewed as a merit since a good thermal conductivity is required for a high performance magnetic refrigerant material^45^. Both a rather low residual resistivity and a considerable large residual resistivity ratio (*RRR*) = 67 indicate that our crystals are of high quality.

Figure 2(b) shows the magnetization as a function of temperature, *M*(*T*), measured from 2 to 300 K in an applied magnetic field *H* = 1000 Oe, aligned both || and ⊥ the *c*-axis, with a field cooling process. A sharp increase of *M* for both directions at the Curie temperature, *T*_*C*_ ~ 220 K, confirms the occurrence of a ferromagnetic transition. Larger magnetization along *c*-axis suggests that the easy axis of magnetization is in the *c* axis. As discussed by Uhl *et al.*^43^ and Pelizzone *et al.*^44^, the strong magnetic anisotropy observed in the ferromagnetic state is certainly related to its peculiar structure being composed of |Fe_3_Te_3_|_∞_ chains, whose central part is a column of edge-sharing octahedral Fe clusters. The Fe-Fe distance of 2.6 Å within the clusters are comparable to the interatomic distance in metallic iron, while the nearest two Fe atoms belong to different |Fe_3_Te_3_|_∞_ chains are 6.7 Å apart. Thus, a strong anisotropy of the exchange coupling is to be expected. As shown in Fig. 2(c,d), it is clear that the *M*(*T*) curves near *T*_*C*_ exhibit a small thermal hysteresis for both directions, which is in contrast to that reported by Uhl *et al.*^43^ and Pelizzone *et al.*^44^, who did not observe any hysteresis in their measurements. We observed a distinguishable but very small hysteresis, (*i.e.*, the hysteresis temperature Δ*T*_*hy*_ = 0.2 K for *H* || *c*-axis and 0.1 K for *H* ⊥ *c*-axis), which suggests that a first-order ferromagnetic transition occurs at ~220 K.

**Figure 3:**
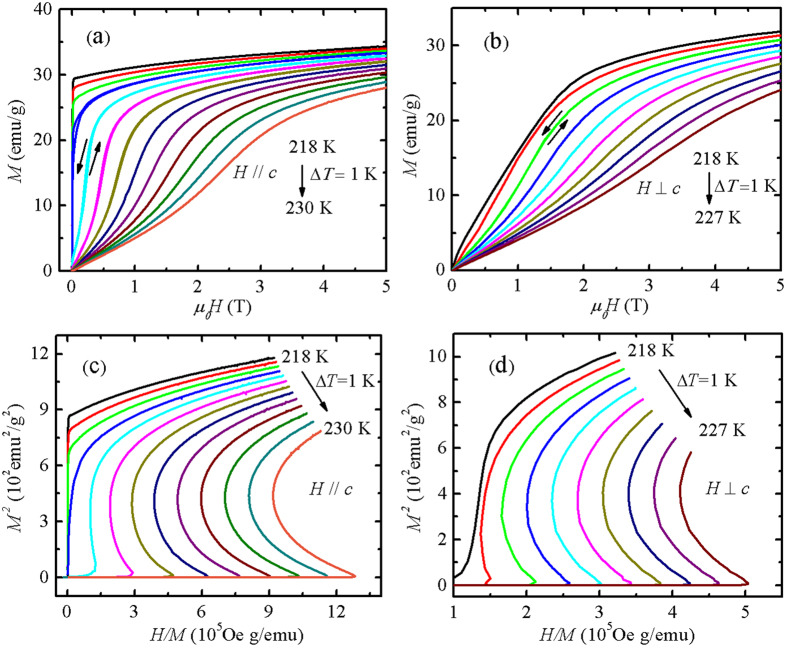
The isothermal magnetization near *T*_*C*_ as a function of magnetic field, *M*(*H*), measured with a temperature step of 1 K for *H* (**a**) || and (**b**) ⊥ the *c* axis. The arrows indicate the measurements with increasing and decreasing magnetic field process. The corresponding *M*^2^ vs *H*/*M* curves for *H* (**c**) || and (**d**) ⊥ the *c* axis.

In order to further identify the type of the transition and to explore the MCE, we performed the isothermal magnetization measurements near the *T*_*C*_. Figure 3 shows the magnetization as a function of magnetic field, *M*(*H*), measured at various temperatures around *T*_*C*_ with both *H* || *c*-axis and *H* ⊥ *c*-axis, and with both increasing and decreasing magnetic field. A small magnetic hysteresis was again observed. The maximum hysteresis is 50 Oe for *H* || *c*-axis [see Fig. 3(a)], while for *H* ⊥ *c*-axis, the hysteresis is rather small and becomes even indiscernible [see Fig. 3(b)]. The *M*(*H*) curves for both *H* || *c*-axis and *H* ⊥ *c*-axis exhibit a different behavior, which is associated with the large anisotropy of magnetization discussed above. The *M*^2^ versus *H*/*M* curves for both directions are shown in Fig. 3(c,d), respectively. According to the Banerjee criterion^46^, the curves at some temperatures have a negative slope and a inflection, which confirms further the occurrence of the first-order ferromagnetic transition around 220 K. The small hysteresis in *M*(*H*) curves enables us to use the Maxwell equation to estimate the isothermal magnetic entropy change (Δ*S*_*M*_). The Δ*S*_*M*_ is calculated by a formula:


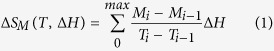


which is an approximation of the integral form of the Maxwell equation.


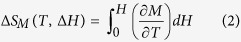


**Figure 4:**
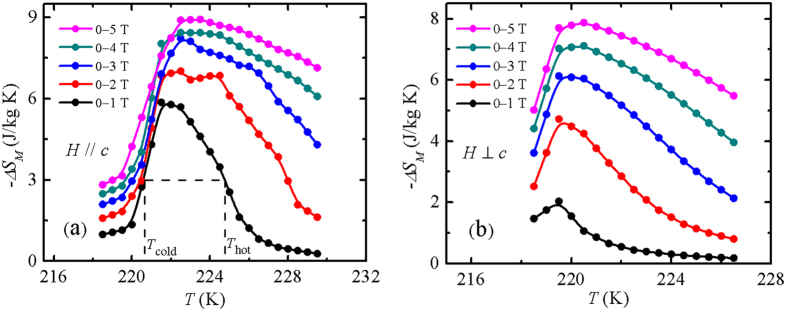
The magnetic entropy change as a function of temperature, −Δ*S*_*M*_(*T*), around *T*_*C*_, with the different field change Δ*H* = 0–1, 0–2, 0–3, 0–4, and 0–5 T for *H* (**a**) || and (**b**) ⊥ the *c* axis. *T*_*hot*_ −*T*_*cold*_ in (**a**) represents the full width at half maximum in −Δ*S*_*M*_(*T*) curve for Δ*H* = 0–1 T.

Figure 4(a,b) present the temperature dependence of −Δ*S*_*M*_ with the magnetic field changes Δ*H* up to 0–5 T, for both *H* || *c*-axis and *H* ⊥ *c*-axis. For *H* || *c*-axis, the −Δ*S*_*M*_(*T*) curve with Δ*H* = 0–1 T shows a pronounced peak around *T*_*C*_, and a table-like behavior can be observed in the −Δ*S*_*M*_(*T*) curves with Δ*H* = 0–2 T and 0–3 T, *i.e.*, there is a temperature range corresponding to the maximum value of magnetic entropy change, which is beneficial for application. With Δ*H* = 0–1, 0–2, 0–3, 0–4, and 0–5 T, −Δ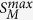 = 5.9, 7.0, 8.2, 8.5 and 8.9 J/kg K, respectively, which increases continuously with the increasing field change and tends to almost saturate at higher magnetic field change. It is known that a “table-like” behavior and no strong Δ*H* dependence of −Δ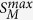 value are the typical behaviors for FOMT materials^2^,^45^. Although −Δ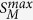 values are smaller than that for the some giant MCE materials (see Table 1), these values of TlFe_3_Te_3_ are comparable with the most potential magnetic refrigerant materials with the a first-order ferromagnetic transition (see Table 1). For the *H* ⊥ *c*-axis case, all the −Δ*S*_*M*_(*T*) curves with different Δ*H* values exhibit a peak around *T*_*C*_ without table-like behavior, and the maximum value of magnetic entropy change −Δ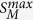 is smaller than that for the *H* || *c*-axis. The anisotropy of MCE may origin from the peculiar magnetic structure, as discussed above.

Another important quality factor of magnetic refrigerant materials is the relative cooling power (RCP) or/and refrigeration capacity (RC), defined^29^ usually as the product of −Δ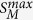 and the full width at half maximum in the −Δ*S*_*M*_(*T*) curve, as an example, *i.e.*, *T*_*hot*_ − *T*_*cold*_ for Δ*H* = 0–1 T in Fig. 4(a). RCP/RC is a measurement of the amount of heat transfer between the cold and hot reservoirs in an ideal refrigeration cycle. Due to the limitation of data measured in our experiments, we only estimated that the RCP values for the Δ*H* = 0–1, 0–2 and 0–3 T, are of 13, 50, and 74.6 J/kg, respectively. Recently, as a figure of merit for the magnetic refrigerant materials, the dimensionless materials efficiency^47^,^48^, *η* = |*Q*/*W*|, is taken into consideration, where electrical or mechanical work, *W*, is done to drive highly reversible caloric effects in an isothermal body, whose entropy is thus modified such that heat, *Q*, flows to (*Q* < 0) or from (*Q* > 0). Here, we estimated the mass-normalized values of |*W*| by integrating −*μ*_0_*MdH*_0_ from the *M*(*H*_0_) data at *T*_*C*_, and evaluated the mass-normalized value of heat *Q* by integrating *μ*_0_*T*_0_(∂*M*/∂*T*)_*H*_ with respect to *H* from the *M*(*H*_0_) data at *T*_*C*_, which follows from the Maxwell relation *μ*_0_(∂*M*/∂*T*)_*H*_ = (∂*S*/∂*H*)_*T*_. The materials efficiency *η* values at *T*_*C*_ was estimated to be of 65.7, 32.0, 23.2, 17.1, and 13.9 for Δ*H* = 0–1, 0–2, 0–3, 0–4, and 0–5 T, respectively.

As a comparison of MCE properties, we choose several compounds with a similar magnetic transition temperature, *T*_*M*_, as well as some typical materials with a near room temperature, *T*_*M*_, focusing on the performence under Δ*H* = 0–2 T (the maximum magnetic field generated by a permanent magnet is about 2 T). As listed in Table 1, although the −Δ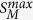 of TlFe_3_Te_3_ is less than that in the some pronounced materials with FOMT, such as GdSi_2_Ge_2_, MnFeP_0.45_As_0.55_, LaFe_11.7_Si_1.3_ and 20-LaFe_11.57_Si_1.43_ materials, −Δ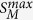 of TlFe_3_Te_3_ is significantly larger than that with SOMT. Both the RCP and *η* values of TlFe_3_Te_3_ are comparable with the most MEC materials, except for some special compounds, such as Tb_5_Si_4_, LaFe_11.7_Si_1.3_, GdSi_2_Ge_2_ and MnFeP_0.45_As_0.55_. Besides having a larger ΔS_*M*_, TlFe_3_Te_3_ has some other advantages, such as a rare-earth-free element, a low synthesis temperature, as well as a low-level hysteresis in the as-grown crystals. But it should be pointed out that the toxicity of Tl element is not so good for the commercial utilization, which may be improved by the replacement of In, Ba, K for Tl in the future.

In summary, after successfully growing TlFe_3_Te_3_ single crystals, we carried systematically out the measurements of its resistivity and magnetization to investigate the nature of the magnetic phase transition and the MCE. It was found that TlFe_3_Te_3_ is a FOMT metal with *T*_*C*_ = 220 K and has a small thermal and field hysteresis near T_*C*_. The relative large MCE at a low Δ*H* makes this compound a promising candidate for magnetic refrigeration around 220 K. Further efforts should be done to substitute Tl by other nontoxic elements in order to utilize this type of materials widely.

## Methods

Single crystals of TlFe_3_Te_3_ were grown using a self-flux method. A mixture with a ratio of Tl:Fe:Te = 1:3:3 was placed in an alumina crucible, sealed in an evacuated quartz tube, heated at 923 K for 5 days. The product was a black powder from which needle-like single crystals with a typical dimension of ~0.4 × 0.4 × 4 mm^3^ could be isolated. Powder XRD measurements on crushed single crystals were carried out at room temperature on a PANalytical x-ray diffractometer (Model EMPYREAN) with a monochromatic Cu *K*_*α*1_ radiation to identify the phase purity and the crystal structure. The composition was confirmed by an electron probe micro-analyzer (EPMA) (Jeol JXA-8100). The magnetic measurements were performed on a Quantum Design Magnetic Property Measurement System (SQUID-VSM, MPMS-5) and the resistivity measurements were carried out on a Physical Property Measurement System (PPMS-9).

## Additional Information

**How to cite this article**: Mao, Q. *et al.* Large low field magnetocaloric effect in first-order phase transition compound TlFe_3_Te_3_ with low-level hysteresis. *Sci. Rep.* **6**, 34235; doi: 10.1038/srep34235 (2016).

## Figures and Tables

**Table 1:** Comparison of the MCE properties with some representative materials with a similar magnetic transition temperature.

Sample	*T*_*M*_	−Δ 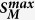 (0–2 T)	Δ*^Thy^*	RCP (0–2 T)	*η* (0–2 T)	Transition type	Ref.
TlFe_3_Te_3_	220	7.02	0.2	50.4	32.0	FOMT	This work
TbCo_2_	231	3.52	0	82.7	11.0	SOMT	49
Gd_2_In_0.8_Al_0.2_	198	3.0	0	31.2	7.29	SOMT	50
Tb_5_Si_4_	225	5.2	0	205.4	—	SOMT	51
LaFe_11_(Si_0.5_Al_0.5_)_2_	213	3.7	0	—	8.1	SOMT	52
Ni_50_Mn_34_In_16_	190	9.5	~8	93.1	36.6	FOMT	48, 53
LaFe_11.7_Si_1.3_	184	28	~1	540	37.6–50	FOMT	48
20-LaFe_11.57_Si_1.43_	198	11.1	3	66.8	23.4	FOMT	39
40-LaFe_11.57_Si_1.43_	210	5.4	0.4	60.2	7.8	FOMT	39
GdSi_2_Ge_2_	276	14	2–14	142	27.2	FOMT	6, 48
MnFeP_0.45_As_0.55_	308	14.5	>1	150	96.7	FOMT	17, 48
Ni_50_Mn_37_Sn_13_	299	6.9	—	96.6	66.8	FOMT	48, 54

The 20-LaFe_11.57_Si_1.43_ and 40-LaFe_11.57_Si_1.43_ represents the ribbon samples prepared at 20 m/s and 40 m/s rates, respectively. The units of *T*_*M*_ and Δ*T*_*hy*_ are Kelvin (K), Δ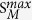 is J/kg K, RCP is J/kg.
